# Health-Related Quality of Life Among People Diagnosed with High-Risk Non-Muscle Invasive Bladder: Baseline Data from the ANZUP BCG + Mitomycin Clinical Trial

**DOI:** 10.3390/cancers18182923

**Published:** 2026-09-09

**Authors:** Kathryn Schubach, Theo Niyonsenga, Carla Thamm, Dickon Hayne, Catherine Paterson

**Affiliations:** 1 Caring Futures Institute, College of Health and Enablement, Flinders University, Adelaide, SA 5001, Australia; carla.thamm@flinders.edu.au (C.T.); catherine.paterson@flinders.edu.au (C.P.); 2 Faculty of Health, University of Canberra, Bruce, ACT 2617, Australia; theo.niyonsenga@canberra.edu.au; 3 Urology Department, University of Western Australia, Crawley, WA 6009, Australia; dickon.hayne@uwa.edu.au; 4 Central Adelaide Local Health Network, Adelaide, SA 5001, Australia

**Keywords:** high-risk non-muscle invasive bladder cancer, health-related quality of life, patient-reported outcomes, supportive care, intravesical therapy

## Abstract

**Background/Objectives:** Intravesical therapy is associated with a range of side effects. This study assessed baseline self-reported health-related quality of life (HRQoL) scores and examined associations with clinical and demographic variables, as well as lower urinary symptom severity and bother scores, among individuals diagnosed with high-risk non-muscle invasive bladder cancer (NMIBC) prior to initiating intravesical Bacillus Calmette–Guérin (BCG) therapy, either alone or in combination with Mitomycin. **Methods**: Baseline data were collected from 501 participants with high-risk NMIBC enrolled in the Australian and New Zealand Urogenital Clinical Trials Group (ANZUP) BCG + Mitomycin Clinical Trial, of whom 438 completed the European Organisation for Research and Treatment of Cancer (EORTC) QLQ-C 30, QLQ-NMIBC 24, and the International Prostate Symptom Scoring Tool (IPSS) prior to commencing intravesical treatment. We conducted descriptive and multivariate statistical analyses. We used hierarchical linear regression to examine associations between HRQoL (Global Health Status) and physical functioning, emotional functioning, sexual function, sexual intimacy, and the IPSS total and bother scores. **Results:** Participants were predominately male (82%), with a mean age of 74.9 years. Over half reported moderate urinary symptoms that affected quality of life. Emotional functioning (β = 0.411, *p* < 0.001) and physical functioning (β = 0.355, *p* < 0.001) were the strongest predictors of HRQoL after adjusting for covariates. **Conclusions:** Individuals diagnosed with high-risk NMIBC report notable physical and emotional concerns prior to commencing intravesical therapy. Targeted interventions may be required to optimise physical and psychological reserve before treatment.

## Simple Summary

People diagnosed with high-risk non-muscle invasive bladder cancer often experience substantial physical and emotional challenges before beginning intravesical therapy. This study reports baseline self-reported health-related quality of life (HRQoL) scores and examines how demographic and clinical factors, as well as lower urinary tract symptoms, relate to HRQoL prior to treatment. Understanding these baseline scores is essential for clinicians, as it enables proactive care planning, supports monitoring throughout treatment, and informs the development of personalised prehabilitation and rehabilitation programs. These insights can help strengthen patients’ physical and psychological resilience before they commence intensive intravesical therapy.

## 1. Introduction

Non-muscle invasive bladder cancer (NMIBC) accounts for approximately 75% of all bladder cancers [1,2,3,4]. Incidence is higher in males than in females, with a ratio of 3:1 [5,6]. People with NMIBC often undergo repeated invasive procedures and either intravesical immunotherapy or chemotherapy, which can negatively affect multiple domains of quality of life [7]. Despite intensive treatment regimens for NMIBC, there is potential for recurrence and progression into muscle-invasive disease, particularly with high-risk disease [8].

NMIBC includes tumours confined to bladder mucosa (Ta) or those invading the lamina propria (T1). Carcinoma in situ (CIS) is a high-grade (HG) tumour that is flat and confined to the mucosa [4]. Historically, NMIBC grading followed the 1973 WHO three-tier system (Grades 1–3), with Grade 3 as the highest grade. The classification was updated in 2004 and 2016 to comprise low-grade (LG) and high-grade (HG) tumours [9]. Current European Association of Urology (EAU) guidelines recommend using both tiers to stage NMIBC [6]. Treatment for high-grade NMIBC typically involves a transurethral resection of the bladder tumour, followed by either adjuvant intravesical chemotherapy or immunotherapy [5,6]. These treatments can significantly affect HRQoL and increase supportive care needs [10].

Evidence regarding self-reported HRQoL and supportive care needs in NMIBC remains limited [2,3,10]. HRQoL is a multidimensional assessment of an individual’s perceived physical, psychological, social, and spiritual well-being in response to NMIBC and its associated treatments. It has been associated with supportive care requirements [11,12]. Supportive care needs are defined as the requirements of an individual or family member for assistance with a particular issue throughout their cancer trajectory [13]. Supportive care provision may address a broad range of care needs, including physical, emotional, spiritual, social and informational needs [13,14]. Evidence indicates a causal relationship between reduced HRQoL and increased supportive care needs [15,16].

Prior research has identified physical concerns, emotional concerns, and urinary symptoms as significant unmet needs among people with NMIBC [17]. This evidence informed the statistical analysis of baseline HRQoL and its associations with demographic variables and lower urinary symptoms in the present quantitative study.

Understanding baseline HRQoL before intravesical therapies is clinically important [18]. It enables anticipatory care planning, supports monitoring of treatment-related changes, and informs the development of prehabilitation models tailored to NMIBC [19,20,21].

While previous studies examined HRQoL during survivorship [3], little is known about HRQoL immediately after diagnosis and before intravesical therapy. This study addresses this gap by reporting baseline HRQoL and examining associations with demographic, clinical, and urinary variables.

## 2. Materials and Methods

### 2.1. Study Participants

**Figure 1:**
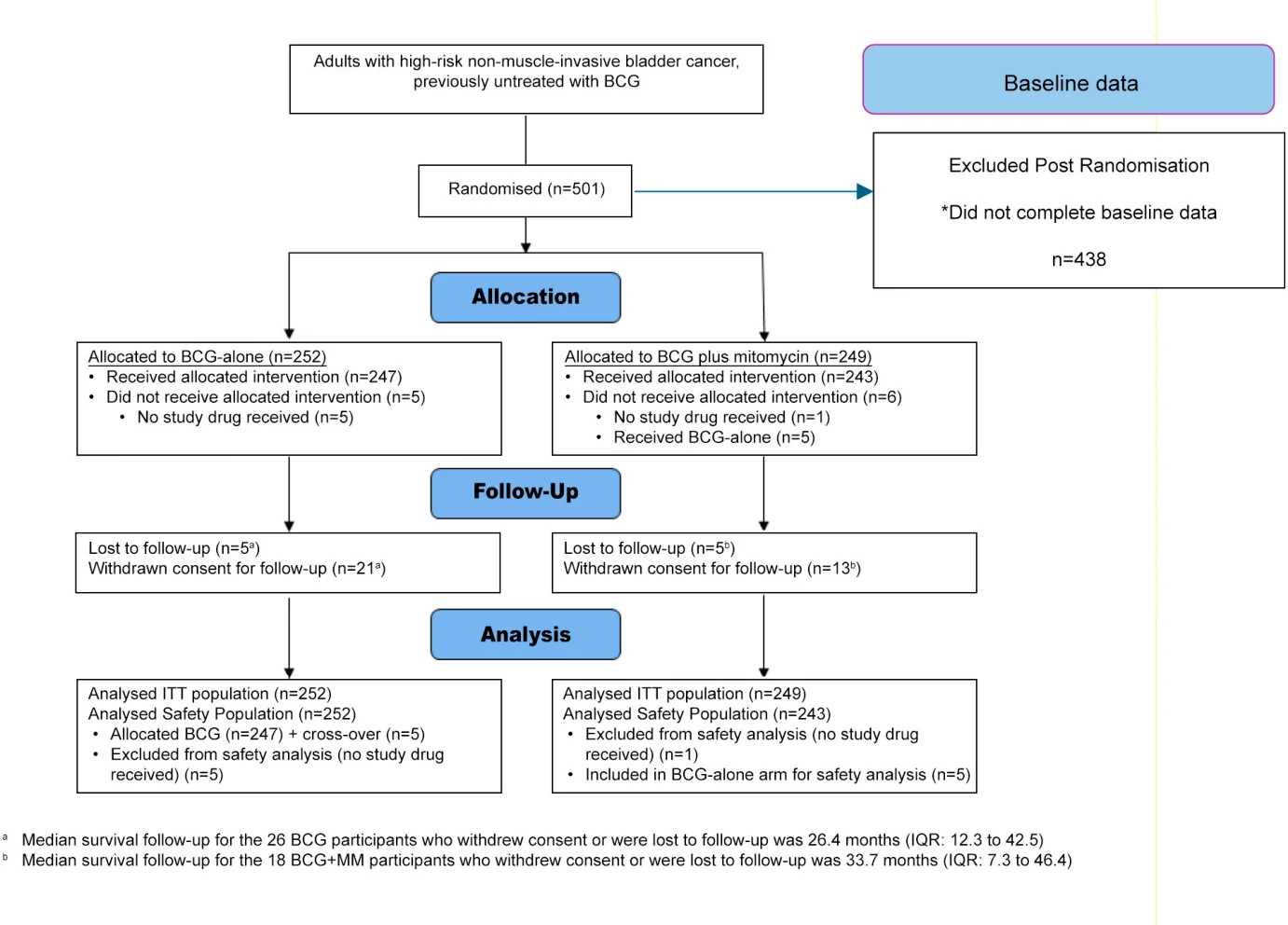
CONSORT Diagram—showing recruitment and total participants in current study. CONSORT Diagram—reproduced and adapted with permission from Prof. Dickon Hayne Chief Investigator (ANZUP 1301) [23]. Copyright licence “CC BY 4.0”.

This secondary analysis used baseline data from the ANZUP BCG + Mitomycin (ANZUP 1301), a phase III randomised trial evaluating the addition of mitomycin to BCG for high-risk NMIBC (see Figure 1 CONSORT diagram) [22]. Eligible participants were adults with confirmed high-grade Ta or any T1 disease, including carcinoma in situ (CIS), who provided informed consent and completed baseline HRQoL questionnaires within 28 days before the first intravesical instillation. Of the 501 enrolled participants, 438 completed HRQoL measures and were included in this analysis. Recruitment occurred between December 2013 and May 2023 across multiple Australian centres and one UK site. Full eligibility data are available in the published protocol [23]. The trial is registered with the Australian New Zealand Clinical Trials Registry (ACTRN12613000513718). The trial protocol permitted a single immediate post-operative instillation of intravesical chemotherapy following TURBT.

### 2.2. Data Collection and Storage Procedures

Secondary baseline data were obtained from ANZUP (1301). The chief investigator and primary study team determined the original sample size and power during the trial’s initial design. The planned sample size was 500 participants (250 per arm) [23]. Participants were centrally randomised to BCG + MM in a 1:1 ratio, stratified by T-stage (pTa-pT1), presence of carcinoma in situ (CIS), and study site. We strictly implemented data governance procedures to ensure compliance with Flinders University’s ethical standards for analysing this baseline clinical trial dataset. The team signed a confidentiality and data governance agreement outlining responsibilities for data handling, storage, and sharing. Ethics approval for the study was granted by the Flinders University Human Research Ethics Committee. This approval confirmed that participant privacy and data protection measures adhered to established ethical and legal standards. All data were securely stored in password-protected systems accessible only to authorised team members, in line with Flinders University’s data management policy.

### 2.3. Measures

Demographics included age, gender, country of birth, and primary language spoken at home. The clinical variables included cancer stage and Eastern Cooperative Oncology Group Performance Status (ECOG). Following randomisation, participants completed three validated instruments: two European Organisation for Research and Treatment of Cancer-(EORTC) QLQ-C30, comprising five functional sub-scales (physical, role, cognitive, emotional, and social), nine symptom scales/items (fatigue, pain, nausea/vomiting, dyspnoea, insomnia, appetite loss, constipation/diarrhoea), along with a financial difficulties scale and a Global Health Status, HRQoL sub-scale. The Global Health Status score is calculated from questions 29 and 30 and then linearly transformed to a scale from 0 to 100. [24] EORTC QLQ-NMIBC24 (formerly known as EORTC QLQ-BLS24), a 24-item survey used in conjunction with the EORTC QLQ-C30. The QLQ-NMIBC 24 assesses symptoms of urinary, bowel, and sexual functioning, yielding a total score ranging from 24 to 96. The higher the score, the greater the symptom burden [25]. The International Prostate Symptom Scoring Tool (IPSS) for the assessment of urinary symptoms. It was initially developed to evaluate lower urinary tract symptoms in men with prostate enlargement [26]. It is a widely used and validated instrument for men and women and has been translated into several languages [27,28,29]. The IPSS contains seven questions that assess urinary symptoms over the past four weeks, including frequency, urgency, nocturia, weak stream, hesitancy, intermittent urinary stream, and incomplete bladder emptying. Item responses are scaled from 0 to 5 (0 = no bother, 5 = great bother), yielding a total score ranging from 0 to 35. Higher scores indicate greater symptom burden [26]. All of these were completed before treatment commenced (up to 28 days before).

### 2.4. Data Analysis

We used descriptive statistics to examine the participants demographics, clinical characteristics, and HRQoL [30]. Independent *t*-tests were conducted to investigate gender differences in HRQoL (global health status) scores and to compare EORTC QLQ-C30 and EORTC BLS-24 QoL symptoms, particularly emotional and physical functioning, which have been previously reported as frequently unmet supportive care needs in the NMIBC population and were considered important independent variables in the statistical analysis [17]. We used Levene’s test to assess whether variances were unequal. We performed a Welch test to assess equality of means across groups [31]. We performed one-way analyses of variance (ANOVAs) to examine the association between HRQoL (global health status) outcomes and selected categorical variables. We conducted a one-way ANOVA with Welch’s correction to compare HRQoL between ECOG status 0 and ECOG status 1 [30]. The assumption of homogeneity of variances was violated, justifying the use of the Welch robust test. Pearson correlations assessed bivariate associations. Hierarchical linear regression was performed to evaluate predictors of HRQoL (global health status), using the variables physical functioning, emotional functioning, sexual function, sexual intimacy, and the total and bother scores from the International Prostate Symptom Score [31].

Comparisons across HRQoL domains are descriptive and hypothesis-generating; the hierarchical regression was the pre-specified inferential analysis. We did not adjust for multiplicity in the descriptive comparisons. We interpreted differences against established thresholds for clinical meaningfulness of 5-point score changes and drew no substantive conclusions from statistical significance alone. Predictors were specified a priori on clinical grounds and entered in blocks ordered by anticipated importance rather than by statistical criteria. Physical and emotional functioning were entered first because a systematic review of supportive care needs in NMIBC identified physical symptoms and psychological distress as the most frequently reported unmet needs in this population [17], making them the domains most relevant to the design of a prehabilitation intervention. Sexual functioning and sexual intimacy were entered second as disease-specific concerns known to persist well into NMIBC survivorship [3]. We entered IPSS total and bother scores third to assess whether lower urinary tract symptom burden added explanatory value beyond the EORTC domains already in the model. We analysed the data using IBM SPSS Statistics (Version 30).

### 2.5. Comparison of EORTC QLQ-C30 Scores to Reference Data

To contextualise the study findings, we compared HRQoL scores with three established EORTC QLQ-C30 reference groups. The first reference group comprised Australian normative data (*n* = 1821), which was relevant given 57% of participants in the present study were born in Australia/Oceania. This cohort included 49.3% males and 50.7% females, with 9.5% reporting a previous cancer diagnosis, although cancer type was not specified [32]. The second reference group consisted of the EORTC general population normative dataset (*n* = 7802), which included individuals with various cancer types (52% males, 48% females). This dataset included no specific data for the NMIBC population [33]. The third reference group was a United Kingdom cohort of individuals diagnosed with NMIBC (*n* = 238), consisting of 79% males and 21% females, assessed three months after diagnosis. Of these, 82 participants had received intravesical therapy prior to completing baseline questionnaires [34]. In contrast, we collected baseline data 28 days post-TURBT and before starting intravesical treatment. We calculated mean scores for all QLQ-C30 and QLQ-NMIBC 24 subscales (range 0–100) according to the EORTC scoring manual [23]. We compared HRQoL scores with Australian normative data, EORTC general population values, and UK NIMBC data. We conducted statistical comparisons using MedCalc Software (Version 23.3.7) [25]. Universal reference values are not available for the QLQ NIMBC 24, as this instrument is still undergoing development to establish normative benchmarks for the NMIBC population [35]. We present comparisons with reference populations as unadjusted descriptive benchmarks and interpret them with caution. The present sample was substantially older (mean age 74.9 years) and predominantly male (82%) compared with the Australian and EORTC reference groups, which differ in sex distribution and cancer status. The UK cohort further differed in treatment timing, as many participants had already commenced intravesical therapy. Scores were not standardised for age or sex; therefore, do not infer adjusted estimates of between-group differences. Differences were interpreted using established EORTC QLQ-C30 thresholds for clinical meaningfulness: <5 points (trivial), 5–10 points (small), 10–20 points (moderate) and >20 points (large). We considered differences statistically significant only when the absolute difference was ≥5 points. Absolute differences and their classifications are reported alongside *p*-values [36].

## 3. Results

### 3.1. Sample Characteristics

A total of 438 participants met eligibility criteria and completed baseline HRQOL measures. The sample was predominantly male (n = 357, 82%), with a mean age of 79.4 (SD 11.4). Over half were born in the Australia/Oceania region (n = 252, 57%), and most spoke English as their primary language (n = 388, 89%). Slightly more than half of participants (231, 53%) had Ta disease, and 207 (47%) had T1 disease. The majority (n = 366, 84%) reported a fully active performance status (ECOG 0). Table 1 summarises the clinical and demographic characteristics of the 438 participants included in the analysis.

### 3.2. Health-Related Quality of Life: QLQ-C30: (Global Health Status) and Functioning

We calculated the HRQoL (global health status) score as the mean of questions 29 and 30 and then linearly transformed it to a 0–100 scale. The mean HRQoL was 76.8 (SD = 17.9). Females reported a slightly higher HRQoL score (78.5 vs. 76.4), although this difference was not statistically significant (*p* = 0.35).

Across functioning domains, males reported significantly higher physical (*p* = 0.03), emotional (*p* = 0.04), and cognitive function (*p* = 0.03); role and social functioning did not differ significantly between genders (see Table 2).

Females reported significantly higher fatigue (*p* = 0.02), insomnia (*p* = 0.01), and loss of appetite (*p* < 0.001). We found no significant differences in pain, dyspnoea, or financial difficulties (see Table 2).

### 3.3. Health-Related Quality of Life: Symptoms (EORTC BLS-24 Symptom Domains)

Males reported significantly greater symptom burden in intravesical treatment-related issues (*p* < 0.01) and sexual functioning (*p* < 0.001). Urinary symptoms did not differ significantly between genders (*p* = 0.16), as shown in Table 3.

For sexual intimacy, males had a mean score of 28.1 (SD = 32.3) versus 5.3 (SD = 11.9) for females, favouring males (*p* < 0.001). However, males’ overall sexual function was statistically significantly higher than that of females (M = 27.1, SD = 25.3 for males versus M = 9.8, SD = 18.5 for females; *p* < 0.01), as shown in Table 3.

### 3.4. HRQoL QLQ C-30 Scores and Clinical Significance Scores Change to Reference Groups

When comparing our study group’s HRQoL (global health status) with the reference groups, the average score was 76.8, which was statistically significant compared with the reference group averages for (1) Australia (68.5) and (2) the EORTC (71.2). However, these differences were clinically small, as shown in Table 4.

Across the functional scales, our study showed statistically significant differences in physical (82.5 vs. 83.8 UK reference group 3, *p* < 0.0001), emotional (82.5 vs. 76.3 EORTC reference group 2, *p* < 0.0001), and cognitive (88.4 vs. 82.8 UK reference group 3, *p* < 0.0004) functioning. However, these score changes were clinically small. Role functioning was the only domain that showed a moderate, clinically significant difference, with statistically higher scores than all reference groups (99.8 vs. 88.8 Australian reference group 1; EORTC reference group 2; 83.0 UK reference group 3; all *p* < 0.0001, as shown in Table 4.

Comparison of symptom scores showed that fatigue was the only symptom that differed statistically across all reference groups (18.8 vs. 23.9, Australian reference group 1; 24.1, EORTC reference group 2; 26.1, UK reference group 3; all *p* < 0.0001), However, the clinical significance of these differences was small. Pain scores (13.5) were statistically lower than those of the Australian reference group (21.8) and the EORTC (20.9) reference groups (*p* = 0.0001), but again with small clinical significance. Constipation scores (10.8 vs. 6.7, *p* = 0.0001) were statistically higher than in EORTC reference group 2, although the magnitude of the change was small. Financial difficulties were statistically lower in our study group than in EORTC reference group 2 (9.2 vs. 9.5, *p* = 0.0001), but the 0.3-point mean difference was well below the 5–10-point threshold for minimal clinical importance, as shown in Table 4.

### 3.5. Health-Related Quality of Life—(Global Health Status) and Its Association with Other Variables; Age Group, Birth Continent, ECOG Performance Status

HRQoL varied by age, with participants aged ≥ 85 reporting the lowest scores. Age significantly predicted HRQoL, with each additional year associated with a 1.21-point decrease. Simple linear regression indicated that the model explained a non-significant proportion of the variance in global health scores. *R*^2^ = [−0.006], *F*([1–436]) = [*F* −2.6], *p* = [*p* −0.107], and age was a significant negative predictor of global health scores, B = −0.121 [β = −0.077], *t* (15.1) ([1–436] = [*t* −15.1]), *p* = [*p* = <0.01]. For every one-year increase in age, a participant’s global health status decreased by an average of 1.21 points. (see Table 5). HRQoL did not differ significantly by continent of birth. ANOVA showed no significant differences in global health status across continent groups, F(18, 419) = 0.330, *p* = 0.996. ECOG status was associated with HRQoL, with ECOG 0 participants reporting higher scores than ECOG 1. We used one-way ANOVA with Welch’s correction. Levene’s test (p = 0.002) violated the assumption of homogeneity of variances, justifying the use of Welch’s robust test. HRQoL (global health status) differed significantly between ECOG 0–1 groups, F (1, 88.54) = 12.60, *p* = 0.003 (see Table 5).

### 3.6. Urinary Symptoms—IPSS Scores

Urinary symptom scores were common: males reported higher IPSS than females (9.8 vs. 7.2), and the difference was statistically significant (*p* < 0.001). The eighth question assessed bother. Male participants reported higher bother from urinary symptoms (58.9%) than female participants (32.4%), and this difference was statistically significant (X^2^ = 16.9, df = 1, *p* < 0.001). Higher total IPSS and bother were associated with lower HRQoL.

A bivariate analysis assessed the linear relationship between the IPSS and HRQoL (global health score). Our results indicated that a higher total IPSS was significantly associated with a lower HRQoL (global health status) score (r = −0.282, *p* =< 0.001). Additionally, greater bother was associated with poorer HRQoL (global health status) (r = −0.222, *p* = 0.001). We grouped total IPSS scores into severity categories: mild (0–7) and moderate-to-severe (8–35). We compared severity categories by sex (total participants: 418; females: 77; males: 341). Severity categories differed by gender, with males more likely to report moderate-to-severe symptoms. The distribution of participants’ urinary symptoms was as follows (females 59%, males 41%); moderate-to-severe (females 41%, males 59%). Males had a higher proportion of moderate-to-severe urinary symptoms than females (*p* = 0.03).

### 3.7. Hierarchical Linear Regression

We conducted a hierarchical linear regression on the overall HRQoL (global health status) score, entering physical functioning, emotional functioning, sexual function, and sexual intimacy as these have previously been identified as unmet needs in the NMIBC population [17], and the IPSS total and bother scores as independent variables in blocks. All necessary assumptions, including normality, multicollinearity, and linearity, were met. In the first block, we entered the predictor variables, physical and emotional functioning, into the model. Hierarchical regression showed that physical and emotional functioning explained 39% of HRQoL variance (*R*^2^ = 0.395, *F* (2,292) = 95.2, *p* < 0.001). Results showed a statistically significant model improvement (*p* = 0.005). Additionally, the *R*^2^ change indicates that adding sexual function and sexual intimacy to the first-block model accounts for only 2.2% of the variation in HRQoL (global health status). IPSS total and bother scores did not significantly improve model fit. Indeed, adding the predictor variables IPSS-total and bother increased the total variance explained by 0.8% (*p* = 0.144), as shown in Table 6.

The results of the third block of hierarchical linear regression indicated that the model improvement was not statistically significant (*p* = 0.144). Indeed, adding the predictor variables IPSS-total and bother increased the total variance by 0.8%, indicating that they did not significantly affect quality of life, given the first two blocks of variables in the model.

Keeping model 2 as the final model and examining the effects of various predictors, as captured by the standardised coefficients (Beta), showed that emotional function (β = 0.411) had a slightly stronger effect than physical function (β = 0.355). Sexual intimacy (β = −0.168) had a negative effect on participants’ HRQoL (global health status), and this effect was statistically significant (*p* = 0.009). In this model, sexual function was the only non-significant predictor (β = 0.061, *p* = 0.192). In the third model, the additional individual variables: total IPSS (β = −0.098) and bother (β = −0.005) were not statistically significant in their impact on participants’ HRQoL, as shown in Table 7.

## 4. Discussion

This study provides baseline evidence on self-reported HRQoL (global health status) scores and urinary symptom scores among individuals with high-risk NMIBC prior to commencing an intravesical treatment regimen. The evaluation utilised validated instruments, specifically the EORTC QLQ-C30, EORTC-BLS 24, and the IPSS. Additionally, the study examined associations with (a) clinical variables, (b) demographic factors, and (c) lower urinary symptom severity and bother scores, while controlling for other covariates. The results showed that over half of the sample experienced moderate to severe lower urinary tract symptoms and impacts on sexual function prior to commencing intravesical therapy, underscoring the importance of early adoption of supportive self-management to optimise symptom control, including models such as prehabilitation.

The average overall HRQoL (global health status) score for this study population was 78.5 for females and 76.4 for males, both higher than those reported in other studies [34,37]. However, when we compared gender differences, males scored higher on five functioning scales, namely physical, role, emotional, cognitive, and social, suggesting that they had a higher perceived quality of life than females, as reported in the literature [38,39]. By contrast, Mercieca et al. (2019) reported that females in their study had higher physical, role, and social functioning scores than men [32]. In our study, women reported more fatigue, insomnia, and appetite loss, which could be explained by other issues, such as menopausal symptoms, which may be attributable to the age range of the participants or other comorbidities that were not captured in the data.

Consistent with existing literature, participants reported notable physical and emotional symptoms before treatment initiation [17]. In the present study, female participants had lower emotional function scores on the EORTC QLQ-C30 (76.7, SD 23.5) than male participants (83.3, SD 17.5). The combined emotional function score for all participants was 82.5 (SD 19.9). Emotional function scores were comparable to reference groups but highlight the need for further exploration of psychological symptoms such as anxiety and uncertainty, which are well-documented unmet needs in the NMIBC population [17].

Our study group had a higher overall HRQoL (global health status) score than the reference groups, with a statistically significant difference; however, the magnitude was of small clinical significance. A previous study by Catto et al. [37] investigated the HRQoL of bladder cancer participants and found that they had the lowest HRQoL when compared to prostate and colorectal patients. This population included participants who had received treatment for their bladder cancer, including muscle-invasive bladder cancer patients, which could explain the difference in findings [37]. Age emerged as a significant negative predictor of HRQoL, with older participants reporting lower HRQoL. Previous studies have shown that younger participants tend to have lower quality-of-life scores due to greater financial burden, more frequent treatment visits, and employment disruptions [35,38,40].

Although statistically significant, several differences between our sample and the reference populations were small in absolute terms and fell below accepted thresholds for clinical meaningfulness. Cognitive functioning, for example, differed from the Australian reference sample by 0.4 points (88 vs. 88, *p* = 0012), a difference detectable at this sample size but without clinical consequence. We have therefore confined our interpretation to differences that were both significant and greater than 5 points. On that basis, the differences that warrant comment are as follows: the functional scores for our study group showed statistically significant differences in physical, emotional, and cognitive functioning. However, these score changes were clinically small. The only functioning scale showing a moderate, clinically significant change was role function, which differed significantly from all reference groups. Participants in our study group had lower pain and fatigue scores, which indicated only small clinical significance. The remainder are consistent with an older, predominantly male, trial-eligible cohort scoring similarly to general population norms, which is itself the more defensible reading of these data.

Sexual function and intimacy scores were higher among male participants than in female participants, and males reported more sexual problems than women. These results are consistent with findings in other studies [37]. Our research also revealed that fewer participants completed the sexuality and intimacy questions than any other question, a pattern observed in other studies [3,37]. However, this may be because these questions ask whether participants were sexually active in the past week. Perhaps to fully understand why participants do not answer these questions, further investigation or other research methods, such as qualitative studies, may be needed to capture this data.

Urinary symptoms reported by people diagnosed with NMIBC are common [3,7,17]. In our study, over half of participants had moderate urinary symptoms before starting treatment. Males had higher mean total scores and greater bother than females, both statistically significant. Further analysis revealed an association between higher total and bother scores on the IPSS and lower HRQoL, a pattern also reported in other studies utilising the IPSS [27]. The reduction in HRQoL among participants may be attributed to urinary symptoms and their effects on sleep and socialisation, consistent with findings from a recent systematic review on this population [17].

Hierarchical regression demonstrated that physical and emotional function were the strongest contributors to HRQoL, explaining 39% of the variance. Sexual intimacy contributed a small but significant additional effect, whereas sexual function, urinary symptom severity, and bother did not meaningfully improve model fit. These findings suggest that interventions targeting physical and emotional factors may have the greatest impact on HRQoL in this cohort.

Non-muscle invasive bladder cancer is a chronic health condition, with sexual and urinary symptoms persisting well into survivorship. The arduous treatment regimen and surveillance protocol provide critical time points for clinicians to assess and intervene. To the best of our knowledge, our study has provided baseline data for 438 high-risk NMIBC participants who completed the EORTC QLQ-C-30 and EORTC NMIBC QLQ-24 at baseline. The results from our study have provided evidence that people diagnosed with high-risk NMIBC have both physical and emotional symptoms prior to commencing a rigorous treatment regimen.

Parallel advances in preclinical modelling, including patient-derived organoid biobanks that recapitulate the molecular heterogeneity of bladder cancer and identify differential drug sensitivity between tumours [41,42]. Taken together, the results underscore the importance of early supportive care, including prehabilitation, to optimise physical capacity and psychological resilience before intravesical therapy. Baseline patient-reported outcomes complement emerging biological approaches to personalised intravesical treatment and highlight the need to consider individual functional and emotional reserve when planning care [42].

## 5. Limitations

This study used cross-sectional data, limiting causal inference. Our study’s strengths included 438 participants who completed a suite of valid and reliable questionnaires at baseline, prior to commencing BCG + Mitomycin intravesical treatment. Some participants may have received immediate post-operative intravesical chemotherapy, which could inflate urinary symptoms and reduce HRQoL scores. However, a single immediate post-operative instillation of intravesical chemotherapy was permitted as part of standard care, and we cannot determine from the trial dataset how many participants received it. Any such effect would be expected to bias urinary symptom scores upwards and HRQoL scores downwards. Our estimates should therefore be interpreted as characterising the post-resection, pre-BCG window rather than an untreated disease state. This window is nonetheless the period during which prehabilitation would be delivered and is therefore the clinically relevant point of assessment for this analysis. Limited demographic data e.g., smoking, socioeconomic status, comorbidities) constrained broader contextual interpretation.

Non-completion of HRQoL measures (12.6%) may have introduced bias, as excluded participants could differ systematically from those included. The data-sharing agreement governing this analysis provides access only to participants who contributed patient-reported outcome data, so we could not compare excluded and included participants on demographic or clinical characteristics. Non-completion of patient-reported measures may be associated with poorer health status, distress, or language and literacy barriers; we cannot exclude the possibility that the analysed sample over-represents participants with better functional status, and that baseline HRQoL is modestly overestimated as a result.

Regression analyses were based on participants who completed the sexual function items (67%). We did not impute missing values, as non-completion of these items reflects the instrument’s conditional structure rather than a plausible missing-at-random process, which skews the sample towards younger, more sexually active individuals. Interpret estimates with this in mind. Shared method variance within the EORTC QLQ-C30 may have inflated associations between functioning domains and HRQoL. The model should therefore be interpreted as identifying which functional domains are most closely associated with overall self-rated health in this cohort and as a basis for prioritising intervention targets, rather than as identifying causal determinants of HRQoL. The cross-sectional design precludes any inference about the direction of effect.

## 6. Conclusions

This quantitative analysis addresses a data gap by providing updated figures from Australia and contributes to the global literature. These findings highlight several practical implications for care prior to intravesical therapy. Individualised prehabilitation strategies may help optimise physical capacity and emotional resilience at a time when patients are vulnerable to escalating treatment-related symptoms. Early psychological support should be considered, particularly for patients reporting lower emotional functioning or heightened fatigue and insomnia. Symptom-specific management—especially for urinary and sexual concerns—remains essential, with attention to gender differences and the limitations of current assessment tools. Collectively, these results reinforce the importance of patient-centred care that integrates functional, emotional, and symptom-based needs into treatment planning, ensuring that patients are better prepared for the demands of intravesical therapy.

## Figures and Tables

**Table 1:** Participants’ demographic and clinical characteristics.

Characteristic	n (%)	Mean (SD)
**Age (years)**	-	74.9 (11.4)
**Gender**		
Male	357 (82%)	75.2 (11.2)
Female	81 (18%)	73.6 (11.9)
**Country of birth**		
Australia/Oceania	252 (57%)	-
Asia	23 (5%)	-
Europe	57 (13%)	-
United Kingdom	87 (20%)	-
Africa	4 (1%)	-
North America	6 (2%)	-
South America	5 (1%)	-
Not recorded	4 (1%)	-
**Primary language spoken at home**		
English	388 (89%)	-
CALD	50 (11%)	-
**Cancer stage**		
Ta	231 (53%)	-
T1	207 (47%)	-
* ECOG status		
0	366 (84%)	-
1	67 (15%)	-
2	5 (1%)	-

Cancer Stage: Ta—non-invasive papillary carcinoma; T1—tumour invades subepithelial connective tissue. * ECOG—Eastern Cooperative Oncology Group Performance Status.

**Table 2:** Comparing EORTC QLQ-C30 Scores by Gender.

Scale	Females (n = 81) Mean (SD)	Males (n = 357) Mean (SD)	Mean Difference (95% CI)	*p*-Value
**HRQOL-Global Health Status**	78.5 (19.5)	76.4 (17.5)		0.35
**Function Scales**				
Physical	86.6 (16.1)	90.2 (13.0)	−3.6 (−6.9 to −0.35)	0.03 *
Role	99.8 (0.21)	99.9 (0.18)	−0.1 (−0.63 to 0.03)	0.47
Emotional	76.7 (23.5)	83.3 (17.5)	−6.6 (−11.2 to −2.1)	0.04 *
Cognitive	84.9 (19.6)	89.2 (15.1)	−4.3 (−8.1 to −0.34)	0.03 *
Social	87.8 (20.3)	87.9 (18.6)	−0.1 (−4.7 to 4.5)	0.95
**Symptom Scales**				
Fatigue	23.7 (23.3)	17.9 (17.4)	5.8 (0.7 to 9.8)	0.02 *
Nausea/Vomiting	2.47 (7.0)	2.5 (8.0)	0.0 (−2.0 to 1.8)	0.91
Pain	14.0 (23.7)	13.4 (19.8)	0.6 (−4.4 to 5.5)	0.82
Dyspnoea	10.9 (19.5)	11.0 (19.5)	−0.1 (−4.8 to 4.6)	0.96
Insomnia	32.1 (33.9)	23.0 (28.9)	9.1 (1.9 to 16.3)	0.01 *
Appetite Loss	10.7 (20.9)	4.4 (12.8)	6.3 (2.7 to 9.8)	<0.001 *
Constipation	14.8 (27.3)	9.9 (20.2)	4.9 (−0.4 to 10.1)	0.07
Diarrhoea	7.0 (14.6)	6.5 (15.9)	0.5 (−3.3 to 4.2)	0.81
Financial Issues	7.4 (19.7)	9.7 (20.3)	−2.3 (−7.2 to 2.6)	0.35

CI confidence interval of the difference HRQOL—*p*-value comparing mean score of male–female participants. Function scales (0–100) higher scores indicate better QOL, Symptoms scores (0–100). Higher scores represent greater symptom burden. * Statistically significant.

**Table 3:** EORTC QLQ-NMIBC24 Symptom Domains by Gender.

Scale	Females n = 81	Females M (SD)	Males n = 357	Males MM (SD)	95% CI/D	*p*-Value
**Multi-items**						
Urinary symptoms	79	21.8 (20.0)	352	25.0 (17.6)	−7.5–1.2	0.16
Malaise	79	3.3 (8.5)	356	2.8 (7.5)	−1.3–2.4	0.55
Future worries	79	37.6 (25.1)	352	30.7 (24.3)	0.88–1.28	0.25
Bloating	79	16.3 (19.0)	352	16.2 (19.0)	0.73–10.1	0.22
Flatulence	71	9.8 (18.5)	339	27.1 (25.3)	−23.5–11.0	<0.001 *
**Sexual functioning**						
Male sexual problems			305	27.6 (32.0)	−64.0–25.4	
Female sexual problems	19	21.0 (35.5)			−17–14.2	
**Single Items**						
Intravesical treatment issues	75	11.7 (21.5)	339	27.1 (25.3)	−21.0–9.2	<0.001 *
Sexual intimacy	22	5.3 (11.9)	305	28.1 (32.3)	−36.5–9.1	<0.001 *
Risk of contaminating partner	22	4.5 (21.3)	352	10.4 (17.0)	−13.3–1.6	0.12
Sexual enjoyment	22	4.5 (21.3.)	166	12.6 (22.1)	−17.9–1.7	0.10

* Statistically significant. CI/D—confidence interval of the difference.

**Table 4:** Comparison of study sample HRQoL scores and Clinical Significance scores change to Reference Groups.

	Participants	(Reference Group 1) Australian General Population [32]	(Reference Group 2) EORTC General Population [33]	(Reference Group 3) UK Bladder Cancer Population [34]
**Number of** **Respondents**	n = 438	n = 1821	n = 7802	n = 238
**Global Health** **Status/QOL**	76.8 (17.9)	68.5 (21.5) *p* = <0.0001 * small	71.2 (22.4) *p* = <0.0001 * small	74.4 (17.9) *p* = 0.0964
**Functioning Scales**				
Physical	89.5 (13.7)	89.2 (19.0) *p* = 0.7554	89.8 (16.2) *p* = 0.7061	83.8 (19.57) *p* = <0.0001 * small
Role	99.8 (0.19)	88.8 (23.4) *p* = <0.0001 * moderate	84.7 (25.4) *p* = <0.0001 * moderate	83.0 (26.4) *p* = <0.0001 * moderate
Emotional	82.5 (19.9)	80.9 (23.1) *p* = 0.1819	76.3 (22.8) *p* = <0.0001 * small	82.5 (21.0) *p* = 1.0000
Cognitive	88.4 (16.1)	88.0 (21.9) *p* = 0.0012	86.1 (20.0) *p* = 0.0181	82.8 (21.3) *p* = 0.0004 * small
Social	87.9 (18.9)	90.7 (23.9) *p* = 0.0224	87.5 (22.9) *p* = 0.7198	83.8 (24.4) *p* = 0.0156
**Symptom Scales**				
Fatigue	18.8 (18.7)	23.9 (22.0) *p* = <0.0001 * small	24.1 (24.0) *p* = <0.0001 * small	26.1 (25.0) *p* = <0.0001 * small
Nausea/ vomiting	2.5 (7.8)	4.6 (17.0) *p* = 0.0117	3.7 (11.7) *p* = 0.0340	3.2 (8.2) *p* = 0.2742
Pain	13.5 (20.5)	21.8 (26) *p* = <0.0001 * small	20.9 (27.6) *p* = <0.0001 * small	17.0 (24.2) *p* = 0.0473
Dyspnoea	11.0 (19.5)	11.7 (23.0) *p* = 0.0588	11.8 (22.8) *p* = 0.4717	16.2 (25.3) *p* = 0.0030
Insomnia	24.6 (30.0)	24.4 (30.0) *p* = 0.9003	21.8 (29.7) *p* = 0.0550	24.5 (27.5) *p* = 0.9660
Appetite loss	5.5 (14.8)	8.6 (21.9) *p* = 0.0050	6.7 (18.3) *p* = 0.1778	6.7 (18.3) *p* = 0.3555
Constipation	10.8 (21.8)	9.4 (22.6) *p* = 0.2414	6.7 (18.4) *p* = <0.0001 * small	9.5 (21.3) *p* = 0.4556
Diarrhoea	6.6 (15.7)	5.9 (20.1) *p* = 0.4963	7.0 (18.0) *p* = 0.6488	6.9(17.0) *p* = 0.8178
Financial difficulties	9.2 (20.2)	6.2 9(23.9) *p* = 0.0153	9.5 (23.3) *p* = <0.0001 * small	5.4 (17.8) *p* = 0.0152

Australian, European Organisation for Research and Treatment of Cancer (EORTC), and United Kingdom (UK) scores—Mean value (SD: standard deviation). Significance of score change: 5–10 Small, >10–20 Moderate, >20 Large.* Statistically significant.

**Table 5:** Comparisons of HRQoL (global health status) score with age, continent of birth, and ECOG status.

Group	Count n = 438	Mean (SD)	95% CI/D	F Value Significance
**Age**				
<64	83	77.4 (17.4)	L-U 74.7–97.0	F = 2.6 *p* = <0.01 *
65–74	98	78.2 (15.7)		
75–84	179	77.9 (18.0)		
>85	78	71.9 (20.0)		
**ECOG Status**				
0	366	78.1 (16.8)		F = 12.6
1	72	70.0 (21.7)	0.006–0.065	*p* = 0.003 *
**Continent of Birth**				
Australia/Oceania	252	77.7 (17.4)	0.000–0.000	
Asia	23	76.4 (15.8)		F = 0.330
Europe	57	76.0 (18.2)		
UK	87	75.4 (18.8)		*p* = 0.996
Africa	4	60.4 (24.8)		
North America	6	79.1 (18.1)		
South America	5	71.1 (18.1)		
**Missing Data**	4			

ECOG—Eastern Cooperative Oncology Group Performance Status. * Statistically significant.

**Table 6:** Hierarchical regression models—Model Summary.

Model	R	*R* ^2^	Adjusted *R*^2^	Standard Error of Estimate	Change Statistics				
					R Square Change	F Change	df1	df2	Sig.F Change
1	0.628	0.395	0.391	13.2	0.395	95.0	2	292	<0.001
2	0.646	0.417	0.408	13.0	0.022	5.4	2	290	0.005
3	0.652	0.425	0.413	13.0	0.008	1.95	2	288	0.144

1 Predicators (Constant) Emotional functional scale, Physical functioning. 2 Predicators (Constant) Emotional functional scale, Physical functioning, Standard score sexual function, Standard score sexual intimacy. 3 Predicators (Constant) Emotional functional scale, Physical functioning, Standard score sexual function, Standard score sexual intimacy, IPPS bother, IPPS range.

**Table 7:** Regression coefficients—Dependent variable HRQoL—(global health status).

Model	Variable	B	SE	95% CI				
				LL	UL	β	t	*p*
1	(Constant)	4.38	5.551	−6.541	15.308		0.790	0.430
	Physical function	0.483	0.058	0.368	0.597	0.390	8.265	<0.001 *
	Emotional function	0.360	0.043	0.276	0.443	0.400	8.464	<0.001 *
2	(Constant)	8.106	5.724	−0.316	19.373		1.416	0.158
	Physical function	0.439	0.059	0.323	0.555	0.355	7.433	<0.001 *
	Emotional function	0.370	0.042	0.288	0.453	0.411	6.819	<0.001 *
	Standard score sexual function	0.040	0.031	−0.020	0.101	0.061	1.308	0.192
	Standard score sexual intimacy	−0.066	0.025	−0.115	−0.016	−0.168	−2.624	0.009
3	(Constant)	13.042	6.413	0.420	25.663		2.034	00.43
	Physical function	0.425	0.59	0.308	0.542	0.344	7.166	0.001 *
	Emotional function	0.357	0.042	0.273	0.440	0.396	8.392	<0.001 *
	Standard score sexual function	0.033	0.031	−0.028	0.093	0.050	1.055	0.292
	Standard score sexual intimacy	−0.056	0.026	−0.107	−0.006	−0.106	−2.194	0.029
	Total IPSS	−0.266	0.174	−0.609	0.078	−0.098	−1.523	0.129
	IPSS bother question	−0.181	2.175	−4.461	4.100	−0.005	−0.083	0.934

Notes: IPSS—International Prostate Symptom Score. * Statistically significant.

## Data Availability

Restrictions apply to the availability of these data. Data were obtained from the Australian and New Zealand Urogenital Clinical Trials Group (ANZUP) and are available from the corresponding author (kathryn.schubach@flinders.edu.au) with the permission of the Australian and New Zealand Urogenital Clinical Trials Group (ANZUP).

## Abbreviations

The following abbreviations are used in this manuscript:

HRQoL	Health-related quality of life
NMIBC	Non-muscle invasive bladder cancer
IPSS	International Prostate Symptom Score
